# Observed versus predicted cardiovascular events and all-cause death in HIV infection: a longitudinal cohort study

**DOI:** 10.1186/s12879-017-2510-x

**Published:** 2017-06-12

**Authors:** Giuseppe Vittorio De Socio, Giacomo Pucci, Franco Baldelli, Giuseppe Schillaci

**Affiliations:** 1 From the Department of Medicine, Unit of Infectious Diseases Azienda Ospedaliera of Perugia and University of Perugia, Santa Maria Hospital, Perugia, Italy; 2 Department of Medicine, University of Perugia and Unit of Internal Medicine, “Santa Maria” Hospital, Terni, Italy

**Keywords:** HIV, Atherosclerosis, Framingham risk, Cardiovascular diseases, Mortality

## Abstract

**Background:**

The aim of the study was to assess the applicability of an algorithm predicting 10-year cardiovascular disease (CVD) generated in the setting of the Framingham Heart Study to a real-life, contemporary Italian cohort of HIV-positive subjects.

**Methods:**

The study was an observational longitudinal cohort study. The probability for 10-year CVD events according to the Framingham algorithm was assessed in 369 consecutive HIV-positive participants free from overt CVD enrolled in 2004, who were followed for a median of 10.0 years (interquartile range, 9.1-10.1). Cardiovascular events included myocardial infarction, hospitalized heart failure, revascularized angina, sudden cardiac death, stroke, peripheral arterial disease.

**Results:**

Over 3097 person-years of observation, we observed a total of 34 CVD events, whereas Framingham algorithm predicted the occurrence of 34.3 CVD events. CVD event rate was 11.0/1000 person-years of follow-up. In a receiver operating characteristics curve analysis, Framingham risk equation showed an excellent predictive value for incident CVD events (c-statistics, 0.83; 95% confidence interval, 0.76-0.90). In a multivariable Cox analysis, age, smoking and diabetes were independent predictors of CVD events. All-cause death rate was 20.0/1000 person-years of follow-up (*n* = 62 deaths). Causes of death included liver diseases (18), malignancies (14), AIDS-related (11); cardiovascular (9) and others (10). In a Cox analysis, age, AIDS diagnosis and chronic hepatitis were independent predictors of death.

**Conclusions:**

Observed CVD events in HIV-infected patients were well predicted by Framingham algorithm. Established major CVD risk factors are the strongest determinants of CVD morbidity in an Italian contemporary cohort of HIV-positive subjects. Interventions to modify traditional risk factors are urgently needed in HIV people.

## Background

Atherosclerotic cardiovascular disease (CVD), a leading cause of morbidity and mortality in the general population, is also an increasing concern for the progressively aging HIV-infected population. Patients on antiretroviral therapy (ART) have a long life expectancy and, as a result, are at risk of developing chronic non-communicable diseases. The increased burden of CVD among HIV-infected patients is likely a consequence of both traditional and non-traditional risk factors, such as immune activation and inflammation that may contribute to an accelerated aging process characterized by higher-than-anticipated rates of noninfectious comorbidities [1, 2].

Risk prediction is a cornerstone of strategies for prevention of CVD. The absolute cardiovascular (CV) risk in a single individual is determined by a complex interplay of risk factors including age, family history of CVD, smoking, hypertension, elevated blood lipids, diabetes, and other determinants [3]. Identifying in clinical practice HIV subjects at high CV risk for primary prevention is a relevant issue, but the optimal measure for predicting the CV risk remains controversial. The Framingham risk equation is a calculated measure of CVD risk, which has been validated in the general population [4], and is internationally considered a valuable tool for patient evaluation and management of primary CV prevention. To date, prospective data regarding CVD events from HIV people are lacking, and the ability of available risk charts to predict CVD events in HIV people are still debated. A first report from the D:A:D: study with 5 years of follow-up indicates that the Framingham equation slightly underestimates the risk of MI in subjects receiving ART [5]. An update from D:A:D: study concluded that Framingham model performed well compared to DAD equation for global CVD risk [6]. In contrast a study from Spain showed that Framingham risk equation significantly overestimated ischemic heart events in South-European HIV-infected patients [7]. We assessed the agreement between predicted global CVD risk according to the Framingham equation [4] and observed CVD morbidity in a consecutive series of HIV-positive subjects who were followed up until 10 years. We also evaluated the main predictors of CV events and all-cause death rate over the same time period.

## Methods

As reported in a previous study [8], consecutive adult subjects with documented HIV infection were recruited at the outpatient clinic of the Unit of Infectious Diseases, University of Perugia, Italy, from January to December 2004. All subjects provided informed consent to participate in the study and they were included irrespective of whether they had been receiving antiretroviral therapy. The consecutive adult patients aged between 30 and 74 years, were examined during baseline clinical evaluation for HIV infection. Individuals with a history of coronary or cerebrovascular disease were excluded from the study. For each patient a complete medical history, physical examination, and laboratory evaluation was completed. Blood was drawn after an 8 to 12 h fast to determine serum total cholesterol values, high-density lipoprotein (HDL) cholesterol, triglycerides, blood glucose, CD4 + T-lymphocyte count, and HIV-RNA. Blood pressure was measured by the physicians in the medical center with a mercury sphygmomanometer after patients sat for 10 min or longer at room temperature. Smokers were considered to be those who smoked one or more cigarettes a day. Patients were followed prospectively at the HIV outpatient clinic for a median of 10 years as part of regular medical care. For each patient we estimated baseline CVD risk according to the global Framingham risk equation [4]. We also estimated the expected numbers of major coronary heart disease (CHD) events over the following 10 years based on the risk equations developed by the “Progetto CUORE”, which has been developed specifically for an Italian population [9, 10]. The 10-year risk for cardiovascular mortality was estimated on the ground of the European SCORE (Systematic COronary Risk Evaluation) algorithm [11]. Clinical data were obtained from electronic or paper medical records. Patients were excluded if they did not complete a minimum of 1 year of follow-up. Collected data were analyzed anonymously. The study was approved by the institutional ethics committee (Ethics Committee of the Umbria Region).

### Study outcomes

We defined CVD events, according Framingham study [4], as a composite of myocardial infarction, hospitalized heart failure, revascularized angina, sudden cardiac death, stroke, transient cerebral ischemia, peripheral arterial disease (intermittent claudication). For the subjects who developed a CV event during follow-up, hospital record forms and other available original source documents were reviewed in conference by the investigators, who were unaware of the baseline clinical data of the subjects examined. Source documents were coded by International Classification of Disease, Ninth Revision, Clinical Modification (ICD-9-CM), codes 410-415, 428, 430-441. We made any effort to carefully identify CV outcome events to avoid potential sources of bias. We also recorded all-cause deaths categorized as liver-related, cancer, defined as non-AIDS-defining malignancies, AIDS-related, including all AIDS-defining malignancy, CV, others.

### Statistical analysis

SPSS statistical package, release 22.0 (SPSS Inc., Chicago, Ill) was used for all statistical analyses. Standard descriptive and comparative analyses were undertaken. Statistical testing was performed at the two-tailed a-level of 0.05. Continuous variables were tested to detect substantial deviations from normality by computing the Kolmogorov-Smirnov Z test. The assumption of satisfactory normal distribution was met for all of the examined variables. Differences between the groups with vs without incident CV events were evaluated by Pearson’s χ^2^ test. The Student *t* test and the Mann-Whitney U test were used to assess differences in normally and non-normally continuous variables respectively.

The rates of CV events are presented as the number of events per 100 patient-years. For those subjects who experienced multiple events, survival analysis was restricted to the first event. The effect of prognostic factors on survival was evaluated with the use of the stepwise Cox semiparametric regression model. The assumption of linearity for the Cox model was tested through visual inspection and no violation of proportional hazards was found. For patients without events, the date of censor was that of the last contact with the patient.

We used the χ^2^ goodness-of-fit test to assess agreement between observed and expected. Individual predictive performances for global Framingham risk equation [4] (CVD events), “Progetto CUORE” equation [9, 10] (major coronary heart disease events) and European SCORE algorithm [11] (CV mortality) were evaluated by receiver operating characteristic (ROC) curve analysis, describing areas under curves with their 95% confidence intervals (CI) and comparing them to the null hypothesis (area = 0.5) [12]. An area under the ROC curve of 1.0 indicates perfect classification of cases (future event) and non-cases (future censoring), whereas 0.5 means that the classification is not better than chance.

## Results

**Fig. 1:**
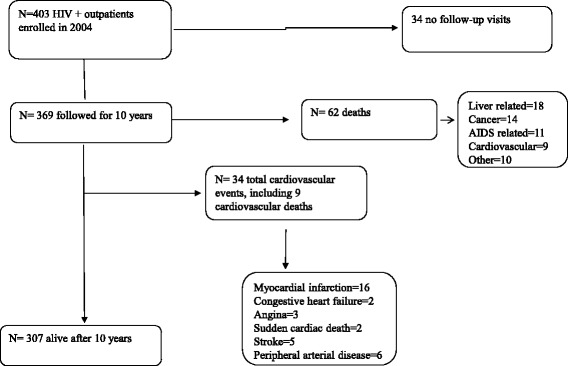
Flow-chart of the study

A total of 403 consecutive HIV outpatients were enrolled. After the exclusion of 34 patients who had no follow-up visits after 1 year, 369 patients were included in the present study. Over a median of ten follow-up years (range, 1-10 years), we observed a total of 34 CV events over 3097 person-years (11.0 CV events/1000 follow-up years). The study flow-chart is reported in Fig. 1.

The main clinical characteristics of the study participants with or without incident events are shown in Tables 1 (CV) and 2 (death). Patients with incident CV events were older (mean age 53.8 ± 12 vs 41.9 ± 8), predominantly male (88.2% vs 60.3), more likely to be smokers (70.6% vs 52.2%), hypertensive on therapy (26.5% vs 7.5%), and diabetic (35.5% vs 4.5%). HIV-related factors such as baseline CDC stage, CD4 cell count, CD4 cell nadir and zenith of HIV-RNA, were not significantly related to incident CV events. As expected, major traditional CV risk factors and CV risk equations were strongly associated with CV events.

**Table 1:** Clinical characteristics at baseline of the study participants with or without incident CV events

	All HIV *n* = 369	CV events *N* = 34	No CV events *N* = 335	Univariate *p*
Age, years	43.0 ± 9	53.8 ± 12	41.9 ± 8	<0.001
Male, *n* (%)	232 (62.9)	30 (88.2)	202 (60.3)	0.001
Follow-up, years (median)	10.0	9.8	10	ns
Body mass index, kg × m^−2^	24.2 ± 4	24.7 ± 5	24.1 ± 4	0.530
Italian “Progetto CUORE” risk, %	3.9 ± 7	13.0 ± 13	2.9 ± 5	<0.001
European SCORE, %	1.3 ± 2	4.2 ± 4	1.0 ± 2	<0.001
Global Framingham CVD risk, %	9.3 ± 11	23.5 ± 17	7.8 ± 10	<0.001
Cigarette smoking, *n* (%)	199 (53.9)	24 (70.6)	175 (52.2)	0.041
Systolic blood pressure, mm Hg	130.5 ± 17	139.7 ± 20	129.5 ± 16	0.008
Diastolic blood pressure, mm Hg	82.3 ± 10	86.5 ± 11	81.2 ± 10	0.024
Pulse pressure, mm Hg	48.2 ± 11	53.2 ± 13	47.7 ± 11	0.020
Treated hypertension, *n* (%)	34 (9.2)	9 (26.5)	25 (7.5)	<0.002
Total cholesterol, mg/dL	180.9 ± 48	174.2 ± 47	181.5 ± 52	0.420
High-density lipoprotein cholesterol, mg/dL	54.6 ± 20	50.6 ± 19	54.9 ± 19	0.218
Statin therapy, *n* (%)	22 (6)	5 (14.7)	17 (5.1)	0.410
Glucose, mg/dL	89.0 ± 23	111.7 ± 46	86.7 ± 18	<0.003
Diabetes, *n* (%)	27 (7.3)	12 (35.3)	15 (4.5)	<0.001
CDC stage C3, *n* (%)	105 (28.5)	14 (41.2)	91 (27.2)	0.084
Baseline CD4 lymphocyte mm^3^	501 ± 309	521 ± 319	500 ± 308	0.714
Baseline HIV-RNA < 50 copies/mL, *n* (%)	256 (69.4)	25 (73.5)	231 (69.0)	0.581
Nadir CD4 lymphocyte mm^3^	186 ± 162	176 ± 158	187 ± 162	0.720
Zenit HIV-RNA, copies/mL (log_10_)	5.0 ± 0.8	5.1 ± 0.9	5.0 ± 0.8	0.336
Hepatitis C infection, *n* (%)	111 (30.2)	13 (38.2)	98 (29.3)	0.282

**Table 2:** Baseline clinical characteristics of the participants, dead vs alive in the follow-up

	All HIV *n* = 369	Dead *N* = 62	Alive *N* = 307	Univariate *p*
Age, years	43.0 ± 9	46.4 ± 8	42.3 ± 9	0.001
Male, *n* (%)	232 (62.9)	44 (71.0)	188 (61.2)	0.148
Body mass index, kg × m^−2^	24.2 ± 4	24.0 ± 4	24.2 ± 4	0.718
IDU risk factor, *n* (%)	101 (27.4)	30 (48.4)	71 (23.1)	<0.001
Cigarette smoking, *n* (%)	199 (53.9)	40 (64.5)	159 (51.8)	0.067
Systolic blood pressure, mm Hg	130.5 ± 17	130.7 ± 20	130 ± 16	0.91
Total cholesterol, mg/dL	181 ± 48	170 ± 47	183 ± 47	0.052
High-density lipoprotein cholesterol, mg/dL	55 ± 20	49 ± 22	56 ± 19	0.218
Diabetes, *n* (%)	27 (7.3)	6 (9.1)	21 (6.8)	0.434
CDC stage C3, *n* (%)	105 (28.5)	32 (51.6)	73 (23.8)	<0.001
Baseline CD4 lymphocyte mm^3^	501 ± 309	393 ± 331	523 ± 300	0.005
Baseline HIV-RNA < 50 copies/mL, *n* (%)	256 (69.4)	41 (46.1)	215 (70.0)	0.543
Nadir CD4 lymphocyte mm^3^	181 ± 158	141 ± 130	189 ± 162	0.012
Zenit HIV-RNA, copies/mL (log_10_)	5.0 ± 0.8	5.0 ± 0.6	5.0 ± 0.8	0.620
Hepatitis C infection, *n* (%)	111 (30.2)	29 (46.8)	82 (26.8)	0.002

Values are mean ± SD

*CVD* cardiovascular disease, *SCORE* systematic coronary risk evalutation, *CDC* Centers for Disease Control and Prevention, *IDU* injecting drug user

**Fig. 2:**
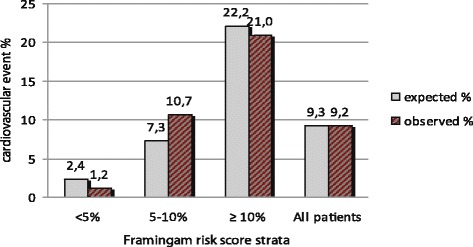
Predicted and observed 10-year cardiovascular event rate by different cardiovascular risk strata (Framingham Risk Score) and in the whole population

The numbers of events predicted by Framingham global risk score [4] and observed in the various categories of CV risk are reported in Fig. 2. CV event rate progressively increased with increasing Framingham Risk Score. As shown, the number of observed CV events (*n* = 34) was well predicted by the Framingham algorithm (*n* = 34.3 events, observed vs predicted *p* = 0.96). Italian “progetto Cuore” estimated a total of 14.3 major CHD events vs 21 observed (6.8 CHD events/1000 follow-up years, observed vs predicted *p* = 0.07), and European SCORE estimated 4.8 fatal CV events vs 9 observed (2.9 fatal CV events/1000 follow-up years, observed vs predicted *p* = 0.053).

**Fig. 3:**
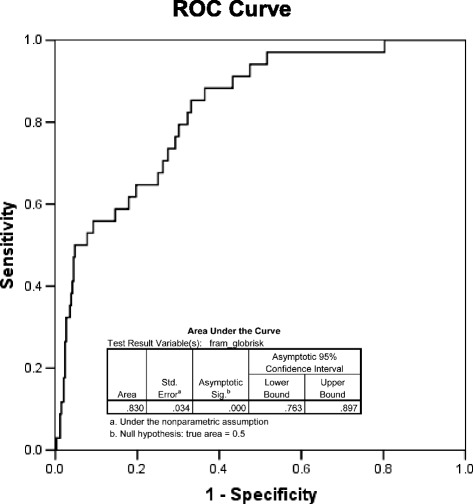
ROC curve analysis receiver operating characteristic (ROC) curve analysis, describing areas under curves with their 95% confidence intervals (CI) and comparing them to the null hypothesis (area = 0.5). An area under the ROC curve of 1.0 indicates perfect classification of cases (future event) and non-cases (future censoring), whereas 0.5 means that the classification is not better than chance

As depicted in Fig. 3, the area under the ROC curve analysis showed that Framingham risk equation was an excellent predictor of CVD events (area under the curve 0.83; 95% confidence interval [CI]: 0.76-0.90). “Progetto Cuore” significantly predicted major coronary events (area under the curve 0.81; 95% CI: 0.72-0.90) and European SCORE predicted cardiovascular death (area under the curve 0.77; 95% CI: 0.67-0.88), although with area values nominally lower than that of Framingham risk equation.

Multivariable analysis using Cox regression (Table 3) showed as significant predictors of incident CV events age, smoking and diabetes. CDC stage and CD4 cells count at baseline had no significantly impact.

**Table 3:** Predictors of incident cardiovascular events

Variable	Hazard ratio (95% CI)	*p*
Age, 1 year	1.10 (1.07-1.15)	<0.001
Cigarette smoking, yes/no	8.6 (3.23-22.88)	<0.001
Diabetes, yes/no	5.143 (2.23-11.83)	<0.001

Multivariate Cox model. BP (or antihypertensive treatment), sex, baseline CD4+ cell count, (or CD4 Nadir), Zenit of HIV-RNA, HCV co-infection failed to enter the final equation.

As regarding all-cause mortality in study population, crude all-cause death rate was 20.0/1000 person-years of follow-up (*n* = 62 deaths). The leading causes of death were respectively liver diseases (18), as non-AIDS-defining malignancies (14), AIDS-related causes (11); cardiovascular (9) and others (10) (Fig. 1). Univariate analysis (Table 2) showed that global mortality was associated whit older age (mean age 46.4 ± 8 vs 42.3 ± 9, *p* < 0.001), CDC stage C (51.6% vs 23.8%, *p* < 0.001), low CD4 cell count (393 ± 331 vs 523 ± 300, *p* = 0.005), HCV co-infection (46.8% vs 26.8%, *p* = 0.002) and injecting drug user (IDU) risk factor for HIV infection (48.4% vs 23.1%, *p* < 0.001). Multivariable analysis using Cox regression (Table 4) showed as significant predictors of 10-year death age, CDC C stage, and chronic hepatitis diagnosis.

**Table 4:** Predictors all-cause deaths

Variable	Hazard ratio (95% CI)	*p*
Age, 1 year	1.04 (1.02-1.07)	0.004
AIDS diagnosis, yes/no	2.26 (1.35-3.81)	0.002
Hepatitis C infection, yes/no	2.36 (1.41-3.95)	0.001

Multivariate Cox model. Sex, baseline CD4+ cell count (or CD4 Nadir), baseline HIV-RNA (or zenith of HIV-RNA), drug abuse failed to enter the final equation

## Discussion

In the present study we examined cardiovascular risk in an outpatient population of HIV-infected patients followed in routine clinical care. Over 3097 person-years, we observed a total of 34 incident cardiovascular events. Event rate was 11.0/1000 person-years of follow-up. By applying global Framingham algorithm for cardiovascular risk estimation, which takes into account the role of conventional risk factors only, we found that HIV-infected individuals had very similar rates of observed clinical CV events compared to the expected ones.

The main findings of the present study may be summarized as follows. First, Framingham model showed good discrimination for 10-year CV events prediction in a contemporary Italian HIV outpatient cohort, with an area under the ROC curve of 0.83 (Fig. 3). Therefore, we validated the Framingham CV risk model in an Italian HIV-infected cohort. The model has been so far validated in several populations, and it has been proved to overestimate CVD risks in countries with a low absolute incidence of coronary events, such as Italy [13]. The present study suggests that this could not be true for Italian HIV-infected patients, who appear more similar to the general population of higher-risk countries than to Italian HIV uninfected subjects. The “real life” setting of the present study suggests that the Framingham model may be appropriate and useful in the daily clinical practice. We also evaluated other widely used risk prediction tools. In our hands, Italian “Progetto Cuore” and European SCORE provide numerically lower estimation rates of incident CV events in Italian HIV patients, although the low number of events is a limitation of this study. Other groups very recently investigated the predictive value of Framingham risk score in longitudinal cohorts and reported moderate discrimination ability. Raggi et al. [14] from Modena HIV Metabolic Clinic showed a moderate sensitivity (69%) and specificity (72%) of Framingham model and a better prediction of atherosclerotic cardiovascular disease (ASCVD) by pooled cohort equation (PCE) algorithm proposed by American Heart Association [15]. However, the CV event rate in Modena cohort (4/1000 patient-years) was lower compared with our data and could be hypothetically related to a more accurate adherence to preventive CV measure in this HIV Metabolic Clinic. Thompson-Paul et al. [16] from HOPS American cohort found moderate discrimination of Framingham risk score (C-statistic: 0.66). The performance of ASCVD PCE algorithm was evaluated very recently in a multicenter clinical cohort from USA, it showed adequately discrimination of myocardial infarction risk (C-statistic: 0.75) [17]. The reasons for difference in prediction ability are not completely clear, but may be due to population differences in cohorts and differences in preventive measure adopted in specific clinical setting.

The second main finding is that this study provides evidence that traditional CV risk factors, directly or hypothetically mediated by HIV/ART, place a major detrimental role in the CV events in HIV-infected patients observed in real life, thus they are essential for risk estimation. Smoking habits, largely present in HIV people, remains the leading cause of preventable illness, thus there is the need of new approaches in stopping smoking in the general population [18] and even more in HIV infected people [19]. We did not document any association between HIV-related factors such as baseline CDC stage and CD4 cells counts, likely to small sample size and probably to secondary role played through non traditional risk factors. In our population, the effective increased risk of CVD through HIV/ART factors appeared modest. Although HIV patients are considered at high CV risk, surprisingly they still tend to be undertreated in terms of drugs for CV prevention [20, 21]. The unquestionable role of conventional risk factors in the actually observed CV events should persuade clinicians to accurately monitor the corrigible CV risk factors in HIV people, such as dyslipidemia, diabetes and hypertension. Understanding and optimizing preventive care in HIV patients is essential in maintaining the substantial advances in prognosis for those subjects [22].

A third relevant finding from this paper is that the 10-year global mortality was largely influenced by HIV infection and/or chronic hepatitis, therefore the optimal treatment of HIV, and virus hepatitis is evidently mandatory. The liver disease was clearly the first cause of mortality in the years investigated. The relatively small sample size and the limited number of events precluded an extensive multivariate analysis and discussion of mortality, although assessment of mortality was not a primary objective of the study.

Strengths of the present study include the well characterized population and quite long time of follow-up. There are several limitations to our analysis; first the lack of an HIV negative control group did not allow us to study the association of HIV infection per se with CV events. The limited sample size may have reduced the power of the study to identify clinically relevant predictors. We conduct our study in a single center, thus data may not be generalizable to HIV people. Figure 2 actually shows a difference between expected and observed in moderate risk patients (5-10% Framingham risk score) with the risk score underestimating the event rate suggesting that possibly with a larger sample size this would be of interest. The limited sample size of the study cohort precludes the evaluation of other relevant CV algorithms such as the D:A:D:, predictive in the next 5 tears and ASCVD PCE algorithms predictive of hard CV events only.

## Conclusion

Observed CVD events in HIV-infected patients were well predicted by Framingham algorithm. In risk assessment for cardiovascular disease Framingham algorithm may still be useful in Italian HIV patients. Established major CV risk factors (age, diabetes, and smoking) are the strongest determinants of CV morbidity in an Italian cotemporary cohort of HIV-positive subjects observed in the real life.

The approach to cardiovascular risk reduction is necessarily multifactorial given the multiplicity of risk factors involved. The data emphasizes the importance of clinical interventions aiming at prevention of modifiable traditional CV risk factors.

## Abbreviation

ART: Antiretroviral therapy
ASCVD: Atherosclerotic cardiovascular disease
CHD: Coronary heart disease
CI: Confidence intervals
CV: Cardiovascular
CVD: Cardiovascular disease
IDU: Injecting drug user
PCE: Pooled cohort equation
ROC: Receiver operating characteristic
SCORE: Systematic COronary Risk Evaluation
